# Determine the accuracy of CBCT reconstructed panoramic images in periodontal assessment

**DOI:** 10.1371/journal.pone.0329604

**Published:** 2025-07-31

**Authors:** Megan Blackstock, Markus Mosley, Erika Stevens, Gerard Camargo, Xiaoxi Cui, Wenjian Zhang

**Affiliations:** Department of General Dentistry, East Carolina University School of Dental Medicine, Greenville, North Carolina, United States of America; Ajman University, UNITED ARAB EMIRATES

## Abstract

**Objectives:**

Periodontal probing and intraoral radiography are conventional methods for periodontal assessment. Evaluating periodontium using CBCT-reconstructed multiplanar or cross-sectional views is labor-intensive and time-consuming. The objective of this study is to evaluate the accuracy of CBCT-simulated panoramic projections for periodontal assessment and determine whether a more efficient evaluation method can be developed.

**Methods:**

CBCTs taken at East Carolina University School of Dental Medicine from 2014–2023 were screened. The inclusion criteria were: (1) diagnostic-quality CBCT scans covering maxilla and/or mandible; (2) full-mouth surveys (FMXs) taken within 6 months of the CBCT scans; and (3) clinical attachment loss (CAL) noted in periodontal charting. The cementoenamel junction (CEJ) to alveolar crest distance on mesial and distal surfaces of maxillary and mandibular central incisors, canines, first premolars, and first molars were measured on bitewings/periapical radiographs (BW/PA) and CBCT-simulated panoramic images. CALs were extracted from periodontal chartings. One-way ANOVA followed by Tukey’s HSD post hoc test, Pearson correlation, and Bland Altman analysis were used to analyze the data.

**Results:**

CBCT, BW/PA, and CAL demonstrated strong correlations, with BW/PA and CBCT producing comparable results in periodontal assessments for most sites. For mandibular central incisors and canines, CBCT and PA showed significantly more bone loss compared to CAL values. For the mandibular molars, CBCT revealed significantly more bone loss compared to BW.

**Conclusions:**

CBCT-reconstructed panoramic imaging has proven to be a reliable tool for periodontal assessment. Some discrepancies among the evaluation methods are likely due to periodontal configurations or conditions, operator errors, and the projection geometry of intraoral radiographs.

## Introduction

Periodontitis is an inflammatory disease of the tooth supporting structures that can ultimately result in tooth loss if left untreated [1]. Periodontal disease was the 11th most prevalent condition worldwide and affects 20–50% of the population, according to the Global Burden of Disease Study conducted in 2016 [2]. The prevalence is expected to increase further due to the rapid aging of the global population [3]. Periodontal disease impairs mastication and nutritional intake and has been found to be associated with negative general health outcomes such as cardiovascular and pulmonary disease, diabetes, and preterm birth [4–7]. Many periodontitis patients seek oral care too late—when the teeth are unsalvageable—because the disease is initially asymptomatic and the bone loss is irreversible [8]. Therefore, early diagnosis and treatment of periodontal disease are critical to maintaining the health of the periodontium.

Periodontal probing and intraoral radiographs are standard methods for periodontal assessment [9]. Periodontal probing helps determine the distance from the bottom of a pocket to the cementoenamel junction (CEJ) [10]. Accurate and reproducible periodontal probing is challenging due to various factors, such as the operator’s training and experience, probing force and angle, tip size and shape, gingival inflammation, the presence of subgingival calculus, and patient cooperation [11,12].

Bitewing (BW) and periapical (PA) radiographs are also valuable diagnostic tools in periodontics. They are commonly used to detect alveolar bone loss associated with periodontitis by measuring the CEJ–alveolar crest distance [13]. However, inherent limitations of 2D imaging—such as superimposition and distortion—affect the reliability and reproducibility of periodontal assessments made with intraoral radiography [14,15].

CBCT, an advanced 3D imaging modality, has gained significant popularity in dentistry due to its low cost, low radiation dosage, small footprint, high spatial resolution, short scanning time, and ease of operation [16–18]. Research has investigated the applicability of CBCT in periodontal assessment. It has been shown that CBCT is helpful in delineating craters, furcation involvement, and other intrabony defects such as fenestration and dehiscence [19–21], as well as providing accurate periodontal bone level measurements [22–28]. However, the majority of these studies used reconstructed multiplanar or cross-sectional views for periodontal assessment. In such cases, a series of sagittal [23–25,27], coronal [24,26,28], or cross-sectional [22,27] reconstructions must be created for every single tooth at the mesial and distal surfaces to measure the CEJ–alveolar crest distance, which is labor-intensive and time-consuming. However, on a CBCT-simulated panoramic projection, all the interproximal surfaces of the existing dentition are visible in a single view. Therefore, all CEJ–alveolar crest distances can be measured from one projection without the need to go through multiple reconstructions. If proven valid, efficiency and convenience are potential benefits of CBCT-derived panoramic reconstructions for periodontal assessment. However, few studies have been conducted in this area.

To address the gap in the current literature regarding whether periodontal bone levels can be accurately and efficiently measured on a single CBCT-simulated panoramic view—rather than through the tedious process of performing measurements on numerous multiplanar or cross-sectional reconstructions, as done in most other studies—the authors initiated this investigation. The purpose is to evaluate the suitability of CBCT-simulated panoramic radiography for periodontal assessment. It is hypothesized that CBCT-simulated panoramic projections can provide reliable periodontal measurements, as they preserve true anatomical relationships due to their 3D nature and avoid the limitations associated with conventional panoramic radiography, such as distortion and magnification.

## Materials and methods

### Subject

CBCTs taken at East Carolina University School of Dental Medicine from 2014–2023 were screened. The inclusion criteria were: (1) diagnostic quality CBCT scans covering the maxilla and/or mandible; (2) full-mouth surveys (FMXs) taken within 6 months of the CBCT scans; and (3) clinical attachment loss (CAL) noted in periodontal charting within 6 months of the CBCT scans. The study was performed in accordance with the ethical standards of the institution and with the 1964 Helsinki Declaration and its later amendments. The study was approved by the Institutional Review Board (IRB) of East Carolina University (UMCIRB 23-000162) without requiring informed consent from the subjects. The patients’ charts were accessed between June 1, 2023, and March 31, 2024. Their chart numbers were linked to a code key, which was destroyed after the data was collected and verified.

### Intraoral radiography

The FMXs were captured using Instrumentarium Focus wall-mounted intraoral x-ray units (Instrumentarium Dental Inc., Charlotte, NC, USA) with an XCP receptor-holding device (Dentsply Rinn, Elgin, IL, USA), ScanX intraoral photostimulable phosphor plates (Air Techniques, Melville, NY, USA), and rectangular collimation. The exposure settings were 70 kVp, 7 mA, and an exposure time commensurate with the anatomical location.

### CBCT acquisition

The CBCT scans were acquired at 85 kVp, 6 mA, 8 x 8 cm field of view (FOV) and a 160 µm voxel size with a Sirona Orthophos SL 3D CBCT scanner (Dentsply Sirona Inc., Charlotte, NC, USA), or at 89.8 kVp, 6 mA, 8 x 6 cm FOV and a 200 µm voxel size with an Instrumentarium OP300 CBCT scanner (KaVo Dental Excellence, Biberach, Germany). CBCT images were reconstructed using Anatomage InVivo Dental 3D software version 6 (Anatomage Co., Santa Clara, CA, USA) at a slice thickness of 1 mm and a bit depth of 16. Images were viewed on a 32-inch LG HDR screen (LG Electronics Inc., Seoul, South Korea) with a resolution of 2560 × 1080 pixels under dimly lit conditions.

### Periodontal assessment

For all patients, the periodontal status was evaluated on the mesial and distal surfaces of the maxillary and mandibular central incisors, canines, first premolars, and first molars. Three evaluators—MB, MM, and ES (authors’ initials)—performed the analysis after calibration.

#### CAL.

Periodontal charting in Axium, the school’s electronic patient record system, was consulted to determine CAL using the formula: CAL = probing depth + CEJ/GM. CEJ/GM refers to the distance between the CEJ and the gingival margin, which is a positive value when the gingival margin is apical to the CEJ and a negative value when it is coronal to the CEJ.

#### BW/PA.

**Fig 1:**
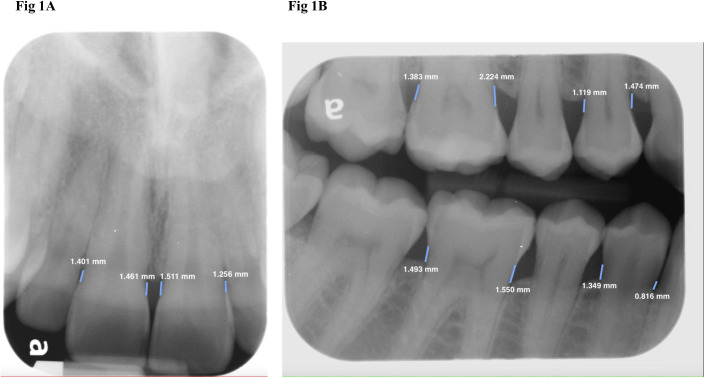
Cementoenamel junction (CEJ) to alveolar crest distance measurements on intraoral radiographs. A. CEJ–alveolar crest distance measurements for maxillary right and left central incisors on periapical radiographs. B. CEJ–alveolar crest distance measurements for maxillary and mandibular first premolars and first molars on bitewing radiographs. The units of measurement are displayed in mm.

FMXs were displayed using Aycan workstation 3.16.010 software (Aycan Medical Systems, Rochester, NY, USA). For central incisors and canines, the CEJ–alveolar crest distance was measured on anterior periapical radiographs using the “ruler” tool (Fig 1A). For first premolars and first molars, the CEJ–alveolar crest distance was measured on bitewings using the same tool (Fig 1B). For teeth with interproximal restorations where the natural CEJ could not be identified, the cervical margins of the restorations were used as the “CEJ.”

#### CBCT.

**Fig 2:**
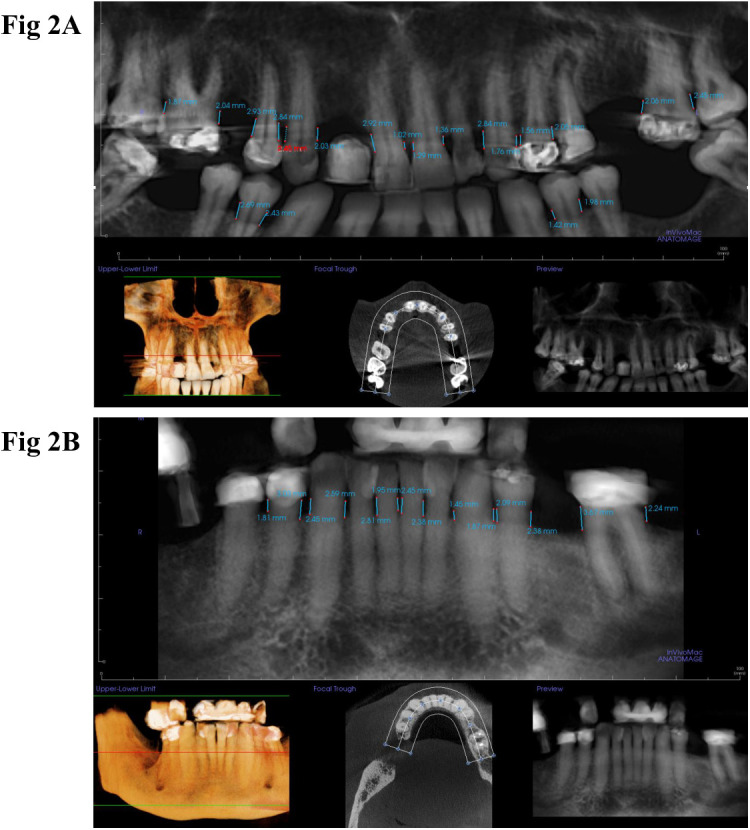
Cementoenamel junction (CEJ) to alveolar crest distance measurements on CBCT-reconstructed panoramic radiographs. A. focal trough was created in the center of the maxillary arch to generate a panoramic projection tailored to the maxilla. CEJ–alveolar crest distances for the maxillary central incisor, canine, first premolar, and first molar were measured on the CBCT-derived panoramic radiograph. B. A focal trough was created in the center of the mandibular arch to generate a panoramic projection tailored to the mandible. CEJ–alveolar crest distances for the mandibular central incisor, canine, first premolar, and first molar were measured on the CBCT-derived panoramic radiograph. The units of measurement are displayed in mm.

CBCT scans were viewed using Anatomage InVivo software. Under the “Panoramic” tab, a focal trough with a fixed width of 10 mm was created in the center of the maxillary or mandibular arch displayed in the axial view, and panoramic reconstructions tailored to the maxilla and mandible were generated. On the reconstructed panoramic views, CEJ–alveolar crest distances were measured on the interproximal surfaces of the aforementioned teeth (Fig 2).

### Statistical analysis

Normal distribution of the data was assessed using the Kolmogorov–Smirnov test. One-way ANOVA followed by Tukey’s HSD post hoc test and Pearson correlation were performed to compare the mean values and analyze correlations among the three groups of data. The three assessment methods were further evaluated using Bland Altman analysis to assess compatibility and potential bias. Intra-class correlation (ICC) was calculated to determine intra-observer and inter-rater reliability, and the results are presented as ICC values with 95% confidence intervals (CIs).

## Results

A total of 48 patients and 768 tooth sites were assessed. The age distribution of the patients ranged from 27 to 84 years, with 27 males and 21 females. The data demonstrated a normal distribution, as confirmed by Kolmogorov–Smirnov analysis. For intra-observer reliability, ICC values were 0.93 (95% CI: 0.88–0.97), 0.81 (95% CI: 0.76–0.85), and 0.87 (95% CI: 0.82–0.92) for MB, MM, and ES, respectively. For inter-rater reliability among the three evaluators, an ICC value of 0.83 (95% CI: 0.75–0.91) was obtained. These results demonstrate good intra-observer and inter-rater reliability for the study.

For maxillary and mandibular central incisors, most interproximal surfaces demonstrated strong correlations among CAL, PA, and CBCT analyses, with comparable results between PA and CBCT. At the distal surface of the mandibular left central incisor and the mesial and distal surfaces of the mandibular right central incisor, PA and CBCT values were significantly higher than those of CAL (Table 1).

**Table 1:** Maxillary and mandibular central incisor CAL, PA and CBCT periodontal assessment.

Tooth/surface	Assessments	N	Mean	SD	P	r	P
**Maxillary right central incisor M**	CAL	37	2.07	0.21		0.74	P < 0.05 (CAL:PA)
	PA	40	2.61	0.26		0.76	
	CBCT	19	2.24	0.13		0.67	
**Maxillary right central incisor D**	CAL	37	2.15	0.24		0.66	P < 0.05 (CAL:PA)
	PA	38	3.08	0.35		0.58	
	CBCT	19	2.43	0.13		0.65	
**Maxillary left central incisor M**	CAL	36	2.17	0.25		0.74	P < 0.05 (CAL:PA)
	PA	37	2.38	0.25		0.74	
	CBCT	19	2.02	3.12		0.57	
**Maxillary left central incisor D**	CAL	36	2.19	0.23		0.68	P < 0.05 (CAL:PA)
	PA	36	2.61	0.29		0.56	P < 0.05 (PA:CB)
	CBCT	18	2.18	0.11		0.56	P < 0.05 (CB:CAL)
**Mandibular left central incisor M**	CAL	40	2.23	0.23		0.64	P < 0.05 (CAL:PA)
	PA	43	9.23	6.38		0.81	
	CBCT	24	3.09	0.15		0.37	
**Mandibular left central incisor D**	CAL	41	2.02	0.22		0.59	P < 0.05 (CAL:PA)
	PA	43	2.75	0.18	<0.05 (PA>CAL)	0.88	P < 0.05 (PA:CB)
	CBCT	23	3.04	0.16	<0.05 (CB >CAL)	0.40	
**Mandibular right central incisor M**	CAL	40	1.99	0.21		0.52	P < 0.05 (CAL:PA)
	PA	42	2.79	0.18	<0.05 (PA>CAL)	0.78	P < 0.05 (PA:CB)
	CBCT	22	2.89	0.16	<0.05 (CB >CAL)	0.34	
**Mandibular right central incisor D**	CAL	41	2.02	0.20		0.53	P < 0.05 (CAL:PA)
	PA	42	2.80	0.20	<0.05 (PA>CAL)	0.67	P < 0.05 (PA:CB)
	CBCT	22	3.12	0.13	<0.05 (CB >CAL)	0.64	P < 0.05 (CB:CAL)

Abbreviations: CAL, clinical attachment loss; PA, periapical radiograph; CBCT, cone beam computed tomography; N, sample size; SD, standard deviation; M, mesial surface; D, distal surface.

In the maxillary and mandibular canine regions, observations similar to those of the central incisors were made: CAL, PA, and CBCT evaluations showed strong correlations, and PA and CBCT produced comparable values for most interproximal sites. At the mesial and distal surfaces of the mandibular left canine, as well as the mesial surface of the mandibular right canine, PA or CBCT demonstrated significantly more bone loss compared to CAL values (Table 2).

**Table 2:** Maxillary and mandibular canine CAL, PA and CBCT periodontal assessment.

Tooth/surface	Assessments	N	Mean	SD	P	r	P
**Maxillary right canine M**	CAL	40	2.44	0.36		0.84	P < 0.05 (CAL:PA)
	PA	40	2.26	0.27		0.73	P < 0.05 (PA:CB)
	CBCT	23	2.66	0.13		0.58	P < 0.05 (CB:CAL)
**Maxillary right canine D**	CAL	40	2.50	0.24		0.51	P < 0.05 (CAL:PA)
	PA	40	2.53	0.21		0.76	P < 0.05 (PA:CB)
	CBCT	23	2.66	0.14		0.66	
**Maxillary left canine M**	CAL	39	2.15	0.23		0.67	P < 0.05 (CAL:PA)
	PA	37	2.14	0.24		0.73	P < 0.05 (PA:CB)
	CBCT	20	2.73	0.21		0.55	P < 0.05 (CB:CAL)
**Maxillary left canine D**	CAL	39	2.23	0.24		0.52	P < 0.05 (CAL:PA)
	PA	36	2.54	0.25		0.9	P < 0.05 (PA:CB)
	CBCT	20	2.90	0.18		0.53	P < 0.05 (CB:CAL)
**Mandibular left canine M**	CAL	42	2.01	0.20		0.6	P < 0.05 (CAL:PA)
	PA	42	2.68	0.19	<0.05 (PA>CAL)	0.59	
	CBCT	24	3.16	0.19	<0.05 (CB >CAL)	0.71	
**Mandibular left canine D**	CAL	42	2.08	0.23		0.75	P < 0.05 (CAL:PA)
	PA	42	2.42	0.19		0.69	
	CBCT	24	3.17	0.25	P < 0.05(CB > CAL)	0.61	
**Mandibular right canine M**	CAL	40	2.05	0.21		0.63	P < 0.05 (CAL:PA)
	PA	40	2.58	0.20		0.48	
	CBCT	24	3.22	0.16	P < 0.05(CB > CAL)	0.67	
**Mandibular right canine D**	CAL	40	2.31	0.30		0.79	P < 0.05 (CAL:PA)
	PA	40	2.52	0.23		0.71	P < 0.05 (PA:CB)
	CBCT	24	3.20	0.18		0.61	P < 0.05 (CB:CAL)

Abbreviations: CAL, clinical attachment loss; PA, periapical radiograph; CBCT, cone beam computed tomography; N, sample size; SD, standard deviation; M, mesial surface; D, distal surface.

For maxillary and mandibular first premolars, CAL, BW, and CBCT periodontal assessments were very similar (P > 0.05), with strong correlations for almost all surfaces (Table 3).

**Table 3:** Maxillary and mandibular first premolar CAL, BW and CBCT periodontal assessment.

Tooth/surface	Assessments	N	Mean	SD	P	r	P
**Maxillary right first premolar M**	CAL	36	2.29	0.22		0.71	P < 0.05 (CAL:BW)
	BW	34	2.41	0.21		0.57	P < 0.05 (BW:CB)
	CBCT	23	2.90	0.21		0.82	
**Maxillary right first premolar D**	CAL	36	2.31	0.18		0.81	P < 0.05 (CAL:BW)
	BW	37	2.50	0.17		0.87	
	CBCT	23	2.78	0.15		0.75	P < 0.05 (CB:CAL)
**Maxillary left first premolar M**	CAL	40	2.48	0.25		0.7	P < 0.05 (CAL:BW)
	BW	37	2.60	0.19		0.65	P < 0.05 (BW:CB)
	CBCT	21	2.80	0.14		0.66	
**Maxillary left first premolar D**	CAL	40	2.54	0.24		0.54	P < 0.05 (CAL:BW)
	BW	39	2.68	0.19		0.6	P < 0.05 (BW:CB)
	CBCT	21	2.86	0.17		0.73	
**Mandibular left first premolar M**	CAL	41	2.07	0.19		0.66	
	BW	38	2.02	0.13		0.56	
	CBCT	26	2.28	0.22		0.74	
**Mandibular left first premolar D**	CAL	42	2.18	0.21		0.58	P < 0.05 (CAL:BW)
	BW	41	2.31	0.16		0.76	P < 0.05 (BW:CB)
	CBCT	26	2.70	0.13		0.58	
**Mandibular right first premolar M**	CAL	41	2.24	0.32		0.7	P < 0.05 (CAL:BW)
	BW	38	2.07	0.22		0.76	P < 0.05 (BW:CB)
	CBCT	23	2.96	0.21		0.73	
**Mandibular right first premolar D**	CAL	24	2.54	0.12		0.58	P < 0.05 (CAL:BW)
	BW	41	2.22	0.21		0.85	P < 0.05 (BW:CB)
	CBCT	24	2.54	0.12		0.81	

Abbreviations: CAL, clinical attachment loss; BW, bitewing radiograph; CBCT, cone beam computed tomography; N, sample size; SD, standard deviation; M, mesial surface; D, distal surface.

In the molar area, periodontal assessments of the maxillary first molars demonstrated strong correlations among CAL, BW, and CBCT, as well as comparable values between BW and CBCT. However, for the mandibular right and left first molars, although strong correlations existed among the three evaluation methods, CBCT revealed significantly more bone loss compared to BW on both interproximal surfaces (Table 4).

**Table 4:** Maxillary and mandibular first molar CAL, BW and CBCT periodontal assessment.

Tooth/surface	Assessments	N	Mean	SD	P	r	P
**Maxillary right first molar M**	CAL	37	3.03	0.24		0.45	P < 0.05 (CAL:BW)
	BW	39	2.73	0.15	P < 0.05(CAL > BW)	0.76	
	CBCT	21	2.89	0.18		0.71	
**Maxillary right first molar D**	CAL	37	3.27	0.28		0.61	P < 0.05 (CAL:BW)
	BW	38	2.70	0.20		0.81	P < 0.05 (BW:CB)
	CBCT	21	3.65	0.26		0.55	P < 0.05 (CB:CAL)
**Maxillary left first molar M**	CAL	34	3.15	0.31		0.43	P < 0.05 (CAL:BW)
	BW	36	2.59	0.24		0.72	P < 0.05 (BW:CB)
	CBCT	20	3.09	0.22		0.78	
**Maxillary left first molar D**	CAL	34	3.71	0.39		0.68	P < 0.05 (CAL:BW)
	BW	35	2.76	0.26		0.85	P < 0.05 (BW:CB)
	CBCT	19	3.54	0.21		0.59	
**Mandibular left first molar M**	CAL	32	2.36	0.22		0.72	P < 0.05 (CAL:BW)
	BW	28	1.89	0.15	P < 0.05(CB > BW)	0.76	
	CBCT	19	2.82	0.13		0.79	
**Mandibular left first molar D**	CAL	32	2.45	0.21		0.43	P < 0.05 (CAL:BW)
	BW	29	2.00	0.13	P < 0.05(CB > BW)	0.86	
	CBCT	18	2.71	0.12		0.57	P < 0.05 (CB:CAL)
**Mandibular right first molar M**	CAL	33	2.53	0.26		0.46	P < 0.05 (CAL:BW)
	BW	31	1.90	0.16	P < 0.05(CB > BW)	0.75	P < 0.05 (BW:CB)
	CBCT	16	2.77	0.11		0.44	
**Mandibular right first molar D**	CAL	33	2.64	0.28		0.8	P < 0.05 (CAL:BW)
	BW	30	2.08	0.14	P < 0.05(CB > BW)	0.78	P < 0.05 (BW:CB)
	CBCT	14	3.39	0.24		0.62	P < 0.05 (CB:CAL)

Abbreviations: CAL, clinical attachment loss; BW, bitewing radiograph; CBCT, cone beam computed tomography; N, sample size; SD, standard deviation; M, mesial surface; D, distal surface.

In summary, the majority of the examined tooth sites exhibited comparable periodontal bone levels as evaluated by CAL, PA/BW, and CBCT, with a few exceptions. In the mandibular central incisor and canine regions, PA or CBCT showed significantly more bone loss compared to CAL values. At the mandibular first molar, CBCT demonstrated significantly more bone loss than the BW assessment. Overall, when all teeth were grouped together, a strong correlation was observed among CAL, PA/BW, and CBCT periodontal assessments (Table 5).

**Table 5:** Summary of correlation analysis.

Assessment	CAL: PA/BW	PA/BW: CBCT	CBCT: CAL
**r value**	0.63	0.73	0.62
**Correlation**	Strong	Strong	Strong

**Fig 3:**
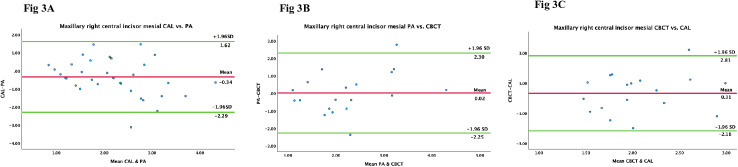
Representative Bland Altman analysis of the mesial surface of the maxillary right central incisor. The plots illustrate comparisons between A. CAL and PA, B. PA and CBCT, and C. CBCT and CAL. The means were close to 0, with most data points randomly scattered around the means and falling within the limits of agreement (95% CI), indicating good agreement among the evaluation methods.

To further confirm the above observations, Bland Altman analysis was performed on the measurements. For most of the tooth sites evaluated—except for certain surfaces of the mandibular anteriors and first molars—the analysis demonstrated good agreement among the three evaluation methods. The associated regression analysis showed no statistically significant differences between the assessments, suggesting no proportional bias and fair compatibility among the three methods (Fig 3).

## Discussion

In the present study, CBCT-derived panoramic radiography was found to be reliable for periodontal assessment, as its results were comparable to PA/BW measurements and showed strong correlations and compatibility with clinical probing and intraoral radiographic evaluations for the majority of the examined dentition sites, as demonstrated by One-way ANOVA, Pearson correlation, and Bland Altman analysis.

Conventional panoramic radiography is not considered the primary imaging modality of choice for periodontal assessment due to its inherent nonuniform magnification, distortion, limited resolution, and the presence of imaging artifacts such as ghost images. Additionally, factors such as upward projection geometry or misalignment of the dentition with the focal trough can obscure visibility of the periodontal apparatus and impair the precision of periodontal evaluation [29–31]. Studies have found that panoramic imaging is not as accurate as intraoral radiographs for assessing periodontal bone loss [32–34].

The present study utilized CBCT-reconstructed panoramic radiography, which represents true anatomical relationships among dental and alveolar structures via a thin slice through the center of the maxillary or mandibular arch. This approach avoids the drawbacks of conventional panoramic radiography. In addition, CEJ–alveolar crest distances can be measured conveniently for all teeth in a single projection, eliminating the need to scroll through a series of sagittal or cross-sectional views to obtain individual tooth measurements [24,27,28]. A fixed focal trough width of 10 mm was used for panoramic reconstruction, as this width has been shown to clearly delineate the CEJ of the examined teeth in the reconstructed panoramic projections.

In the mandibular central incisor and canine regions, CBCT and PA showed significantly more bone loss than CAL values. CAL measures the distance from the CEJ to the base of the periodontal pocket. In contrast, radiographic bone loss measures the distance from the CEJ to the alveolar crest, which theoretically equals CAL plus the biological width. Biological width refers to the distance established from the junctional epithelium and connective tissue attachment to the root surface of a tooth, with an average dimension of 2.04 mm [35]. Therefore, mathematically, radiographic bone loss is expected to be approximately 2 mm greater than the CAL value. In our study, radiographic bone loss was about 1 mm more than CAL in the mandibular central incisor and canine regions, with no significant differences in other tooth locations. This observation could be due to variations in periodontal probing and the periodontal condition itself—especially in periodontitis patients—where increased fragility of the epithelium [36] and degradation of the connective tissue [37] may lead to over-penetration with even slightly elevated probing force, resulting in CAL values that are similar to radiographic bone loss.

For the mandibular first molars, CBCT demonstrated significantly more bone loss compared to BW, which may be partially due to the projection geometry of bitewing radiography. Studies have shown that intraoral radiography tends to underestimate proximal bone levels in periodontal assessment [38,39]. When taking molar BWs, a vertical angulation of +10 degrees is recommended to compensate for the slight bend of the upper portion of the receptor and the tilt of the maxillary teeth. This improves detectability of occlusal caries by minimizing overlap of opposing cusps onto the occlusal surface [40]. However, this oblique angle has been shown to diminish the CEJ–alveolar crest distance measurement, especially for mandibular molars [41–43], which likely accounts for the underestimated periodontal bone levels observed in the current study. In contrast, CBCT preserves the true spatial relationship of these anatomical landmarks.

In the literature, there are inconsistent findings regarding how CBCT periodontal measurements compare to intraoral radiographic examinations and clinical probing. Some studies reported results similar to ours, indicating that radiographic periodontal bone loss appears more severe than CAL values in the incisor region, and that CBCT reveals greater periodontal bone loss compared to intraoral radiographs in the mandibular molar region [23,27,44]. However, other investigations have shown comparable periodontal assessment results between 2D and 3D radiographic examinations [45,46]. Various factors may contribute to these discrepancies, including differences in CBCT scanner types, scanning and reconstruction protocols, evaluation methods, periodontal status, and the experience and training of the examiners.

Despite growing evidence supporting its value in the diagnosis and treatment planning of periodontal disease, CBCT is not considered the first-choice imaging modality, nor should it be routinely used in the management of periodontitis, according to guidelines proposed by SEDENTEXCT [47] and the American Academy of Periodontology [48]. High radiation dose, high cost, and limited accessibility in certain areas or countries are some of the limiting factors for CBCT. Full-mouth radiographic series and clinical probing remain the first-line options for comprehensive periodontal evaluation. CBCT should be judiciously utilized on a case-by-case basis to optimize diagnostic value while minimizing ionizing radiation exposure to the patient [48]. Factors such as metal and beam-hardening artifacts, as well as relatively high radiation doses—especially compared to intraoral radiography—prevent CBCT from being used as a first-line imaging modality in periodontics [49,50]. Although CBCT doses are significantly lower than those of multi-slice medical CT, its effective dose remains several to hundreds of times higher than that of conventional dental radiographs, including intraoral, panoramic, or cephalometric images. This is one of the key reasons CBCT should not be used routinely in periodontal evaluation [51,52].

Although the study was carefully designed, several limitations should be noted. The sample size was relatively small, as few patients met the inclusion criteria during the study period. A future study with a larger sample size will help validate the current findings. Additionally, this investigation was a retrospective study, which allowed for comparisons and correlation analysis among clinical probing, intraoral radiography, and CBCT assessments — but without a true gold standard, which would be intraoperative CEJ–alveolar crest measurements under a reflected flap. Obtaining such a gold standard was not feasible due to the retrospective nature of the study, as well as the likely impermissibility of subjecting most participants to an invasive procedure. The use of secondary data without an intraoperative “gold standard” makes it challenging to fully assess the accuracy and consistency of these evaluation tools. The study was not blinded, as the evaluators could identify intraoral radiographs—periapical/bitewing—or CBCT reconstructions due to their distinct image characteristics. The lack of blinding could introduce bias. Moreover, CAL values were retrieved from older periodontal charting records in Axium, which may contain errors or biases due to factors such as variability in examiner skill, types of periodontal probes used, gingival inflammation status, and calculus distribution. To address the limitations of retrospective studies, a randomized controlled clinical trial incorporating intraoperative periodontal measurements is needed to further corroborate the current observations. Additionally, this study utilized only the Anatomage InVivo software for reconstructing and displaying simulated panoramic projections. Other 3D viewers, such as Planmeca Romexis 3D, Carestream Dental CS 3D Imaging, Dentrix Ascend Imaging, and Blue Sky Plan, should be tested to determine whether similar conclusions can be achieved.

## Conclusions

The CBCT-derived panoramic view has been shown to be an efficient and reliable tool for periodontal evaluation and is considered a valuable addition to clinical probing and intraoral radiography in periodontal assessment.

## Supporting information

S1 Appendix All raw data. (XLSX)

S2 Appendix Summary of one-way ANOVA and Pearson correlation analysis. (XLSX)

S3 Appendix Summary of Bland Altman analysis. (PDF)
