# Supplementary material for: Determine the accuracy of CBCT reconstructed panoramic images in periodontal assessment
Source: PLoS One. 2025 Jul 31;20(7):e0329604. doi: 10.1371/journal.pone.0329604 (PMC12312971; doi:10.1371/journal.pone.0329604)

T-Test

Notes

Output Created		29-APR-2025 13:37:34
Comments		
Input	Active Dataset	DataSet0
	Filter	<none>
	Weight	<none>
	Split File	<none>
	N of Rows in Working Data File	35
Missing Value Handling	Definition of Missing	User defined missing values are treated as missing.
	Cases Used	Statistics for each analysis are based on the cases with no missing or out-of-range data for any variable in the analysis.
Syntax		T-TEST /TESTVAL=0 /MISSING=ANALYSIS /VARIABLES=Difference /ES DISPLAY(TRUE) ...
Resources	Processor Time	00:00:00.01
	Elapsed Time	00:00:00.00

[DataSet0]

One-Sample Statistics

	N	Mean	Std. Deviation	Std. Error Mean
Difference	35	-.3359	.99913	.16888

One-Sample Test

	Test Value = 0					
			Significance			95% Confidence ...
	t	df	One-Sided p	Two-Sided p	Mean Difference	Lower
Difference	-1.989	34	.027	.055	-.33594	-.6792

One-Sample Test

	Test Value = 0
	95% Confidence Interval of the ...
	Upper
Difference	.0073

One-Sample Effect Sizes

		Standardizer[a]	Point Estimate	95% Confidence Interval Lower	95% Confidence Interval Upper
Difference	Cohen's d	.99913	-.336	-.675	.007
	Hedges' correction	1.02187	-.329	-.660	.007

a. The denominator used in estimating the effect sizes.
Cohen's d uses the sample standard deviation.
Hedges' correction uses the sample standard deviation, plus a correction factor.

Regression

Notes

Output Created		29-APR-2025 13:39:25
Comments		
Input	Active Dataset	DataSet0
	Filter	<none>
	Weight	<none>
	Split File	<none>
	N of Rows in Working Data File	35
Missing Value Handling	Definition of Missing	User-defined missing values are treated as missing.
	Cases Used	Statistics are based on cases with no missing values for any variable used.
Syntax		REGRESSION /MISSING LISTWISE /STATISTICS COEFF OUTS R ANOVA /CRITERIA=PIN(.05) POUT(.10) TOLERANCE(.0001) /NOORIGIN /DEPENDENT Difference /METHOD=ENTER Mean.
Resources	Processor Time	00:00:00.02
	Elapsed Time	00:00:00.00
	Memory Required	2480 bytes
	Additional Memory Required for Residual Plots	0 bytes

Variables Entered/Removed[a]

Model	Variables Entered	Variables Removed	Method
1	Mean[b]	.	Enter

a. Dependent Variable: Difference

b. All requested variables entered.

Model Summary

Model	R	R Square	Adjusted R Square	Std. Error of the Estimate
1	.330[a]	.109	.082	.95744

a. Predictors: (Constant), Mean

ANOVA[a]

Model		Sum of Squares	df	Mean Square	F	Sig.
1	Regression	3.690	1	3.690	4.025	.053[b]
	Residual	30.251	33	.917		
	Total	33.941	34			

a. Dependent Variable: Difference

b. Predictors: (Constant), Mean

Coefficients[a]

Model		Unstandardized Coefficients		Standardized Coefficients	t	Sig.
		B	Std. Error	Beta		
1	(Constant)	.551	.471		1.171	.250
	Mean	-.404	.201	-.330	-2.006	.053

a. Dependent Variable: Difference

Notes

Output Created		29-APR-2025 13:45:04
Comments		
Input	Active Dataset	DataSet0
	Filter	<none>
	Weight	<none>
	Split File	<none>
	N of Rows in Working Data File	35
Syntax		GRAPH /SCATTERPLOT(BIVAR)=Mean WITH @8MCAL /MISSING=LISTWISE.
Resources	Processor Time	00:00:00.57
	Elapsed Time	00:00:01.00

Graph

Notes

Output Created		29-APR-2025 13:47:19
Comments		
Input	Active Dataset	DataSet0
	Filter	<none>
	Weight	<none>
	Split File	<none>
	N of Rows in Working Data File	35
Syntax		GRAPH /SCATTERPLOT(BIVAR)=Mean WITH Difference /MISSING=LISTWISE.
Resources	Processor Time	00:00:00.16
	Elapsed Time	00:00:00.00

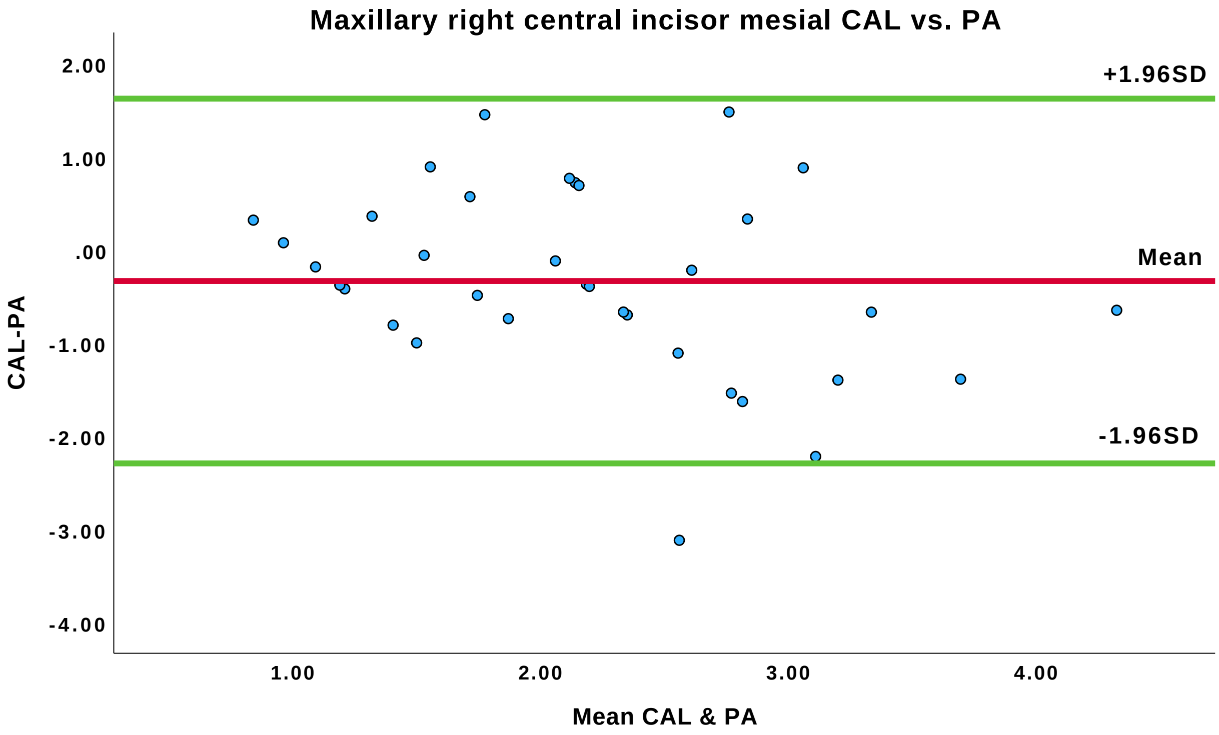
Maxillary right central incisor mesial CAL vs. PA
+1.96SD
Mean
-1.96SD
2.00
1.00
.00
-1.00
-2.00
-3.00
-4.00
CAL-PA
1.00
2.00
3.00
4.00
Mean CAL & PA

T-Test

Notes

Output Created		29-APR-2025 15:19:42
Comments		
Input	Active Dataset	DataSet1
	Filter	<none>
	Weight	<none>
	Split File	<none>
	N of Rows in Working Data File	19
Missing Value Handling	Definition of Missing	User defined missing values are treated as missing.
	Cases Used	Statistics for each analysis are based on the cases with no missing or out-of-range data for any variable in the analysis.
Syntax		T-TEST /TESTVAL=0 /MISSING=ANALYSIS /VARIABLES=Difference /ES DISPLAY(TRUE) ...
Resources	Processor Time	00:00:00.01
	Elapsed Time	00:00:00.00

[DataSet1]

One-Sample Statistics

	N	Mean	Std. Deviation	Std. Error Mean
Difference	19	.0224	1.16137	.26644

One-Sample Test

	Test Value = 0					
			Significance			95% Confidence ...
	t	df	One-Sided p	Two-Sided p	Mean Difference	Lower
Difference	.084	18	.467	.934	.02242	-.5373

One-Sample Test

	Test Value = 0
	95% Confidence Interval of the ...
	Upper
Difference	.5822

One-Sample Effect Sizes

		Standardizer[a]	Point Estimate	95% Confidence Interval Lower	95% Confidence Interval Upper
Difference	Cohen's d	1.16137	.019	-.431	.469
	Hedges' correction	1.21274	.018	-.412	.449

a. The denominator used in estimating the effect sizes.
Cohen's d uses the sample standard deviation.
Hedges' correction uses the sample standard deviation, plus a correction factor.

Regression

Notes

Output Created		29-APR-2025 15:21:35
Comments		
Input	Active Dataset	DataSet1
	Filter	<none>
	Weight	<none>
	Split File	<none>
	N of Rows in Working Data File	19
Missing Value Handling	Definition of Missing	User-defined missing values are treated as missing.
	Cases Used	Statistics are based on cases with no missing values for any variable used.
Syntax		REGRESSION /MISSING LISTWISE /STATISTICS COEFF OUTS R ANOVA /CRITERIA=PIN(.05) POUT(.10) TOLERANCE(.0001) /NOORIGIN /DEPENDENT Difference /METHOD=ENTER Mean.
Resources	Processor Time	00:00:00.02
	Elapsed Time	00:00:00.00
	Memory Required	2528 bytes
	Additional Memory Required for Residual Plots	0 bytes

Variables Entered/Removed[a]

Model	Variables Entered	Variables Removed	Method
1	Mean[b]	.	Enter

a. Dependent Variable: Difference

b. All requested variables entered.

Model Summary

Model	R	R Square	Adjusted R Square	Std. Error of the Estimate
1	.353[a]	.124	.073	1.11827

a. Predictors: (Constant), Mean

ANOVA[a]

Model		Sum of Squares	df	Mean Square	F	Sig.
1	Regression	3.019	1	3.019	2.414	.139[b]
	Residual	21.259	17	1.251		
	Total	24.278	18			

a. Dependent Variable: Difference

b. Predictors: (Constant), Mean

Coefficients[a]

Model		Unstandardized Coefficients		Standardized Coefficients	t	Sig.
		B	Std. Error	Beta		
1	(Constant)	-1.061	.743		-1.428	.171
	Mean	.482	.310	.353	1.554	.139

a. Dependent Variable: Difference

Graph

Notes

Output Created		29-APR-2025 15:25:56
Comments		
Input	Active Dataset	DataSet1
	Filter	<none>
	Weight	<none>
	Split File	<none>
	N of Rows in Working Data File	19
Syntax		GRAPH /SCATTERPLOT(BIVAR)=Mean WITH Difference /MISSING=LISTWISE.
Resources	Processor Time	00:00:00.70
	Elapsed Time	00:00:00.00

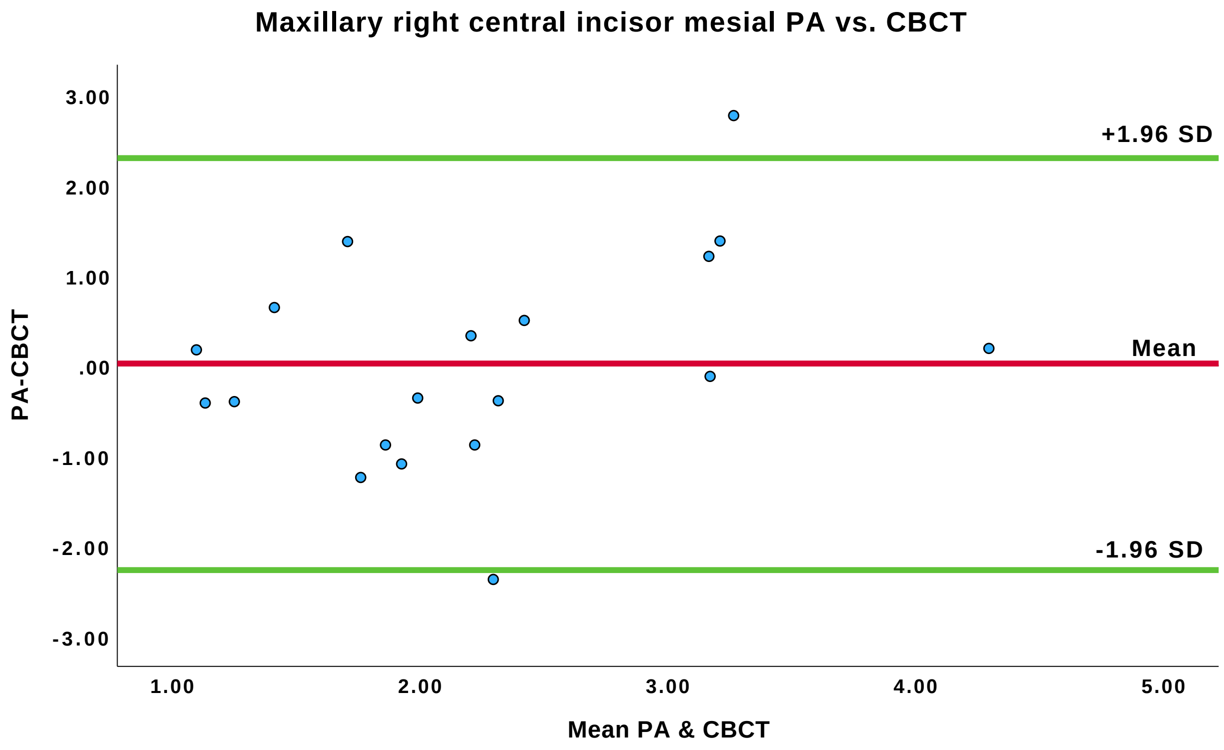

T-Test

Notes

Output Created		29-APR-2025 15:51:00
Comments		
Input	Active Dataset	DataSet0
	Filter	<none>
	Weight	<none>
	Split File	<none>
	N of Rows in Working Data File	19
Missing Value Handling	Definition of Missing	User defined missing values are treated as missing.
	Cases Used	Statistics for each analysis are based on the cases with no missing or out-of-range data for any variable in the analysis.
Syntax		T-TEST<br>/TESTVAL=0<br>/MISSING=ANALYSIS<br><br>/VARIABLES=Difference<br>/ES DISPLAY(TRUE) …
Resources	Processor Time	00:00:00.01
	Elapsed Time	00:00:00.00

[DataSet0]

One-Sample Statistics

	N	Mean	Std. Deviation	Std. Error Mean
Difference	19	.3147	1.27075	.29153

One-Sample Test

	Test Value = 0					
	t	df	Significance One-Sided p	Significance Two-Sided p	Mean Difference	95% Confidence … Lower
Difference	1.080	18	.147	.295	.31474	-.2977

One-Sample Test

	Test Value = 0 95% Confidence Interval of the … Upper
Difference	.9272

One-Sample Effect Sizes

		Standardizer[a]	Point Estimate	95% Confidence Interval	
				Lower	Upper
Difference	Cohen's d	1.27075	.248	-.212	.701
	Hedges' correction	1.32696	.237	-.203	.671

a. The denominator used in estimating the effect sizes.
Cohen's d uses the sample standard deviation.
Hedges' correction uses the sample standard deviation, plus a correction factor.

Regression

Notes

Output Created		29-APR-2025 15:51:44
Comments		
Input	Active Dataset	DataSet0
	Filter	<none>
	Weight	<none>
	Split File	<none>
	N of Rows in Working Data File	19
Missing Value Handling	Definition of Missing	User-defined missing values are treated as missing.
	Cases Used	Statistics are based on cases with no missing values for any variable used.
Syntax		REGRESSION /MISSING LISTWISE /STATISTICS COEFF OUTS R ANOVA /CRITERIA=PIN(.05) POUT(.10) TOLERANCE(.0001) /NOORIGIN /DEPENDENT Difference /METHOD=ENTER Mean.
Resources	Processor Time	00:00:00.02
	Elapsed Time	00:00:00.00
	Memory Required	2560 bytes
	Additional Memory Required for Residual Plots	0 bytes

Variables Entered/Removed[a]

Model	Variables Entered	Variables Removed	Method
1	Mean[b]	.	Enter

a. Dependent Variable: Difference

b. All requested variables entered.

Model Summary

Model	R	R Square	Adjusted R Square	Std. Error of the Estimate
1	.177[a]	.031	-.026	1.28695

a. Predictors: (Constant), Mean

ANOVA[a]

Model		Sum of Squares	df	Mean Square	F	Sig.
1	Regression	.910	1	.910	.550	.469[b]
	Residual	28.156	17	1.656		
	Total	29.067	18			

a. Dependent Variable: Difference

b. Predictors: (Constant), Mean

Coefficients[a]

Model		Unstandardized Coefficients		Standardized Coefficients	t	Sig.
		B	Std. Error	Beta		
1	(Constant)	-.716	1.422		-.504	.621
	Mean	.496	.669	.177	.741	.469

a. Dependent Variable: Difference

Graph

Notes

Output Created		29-APR-2025 15:55:46
Comments		
Input	Active Dataset	DataSet0
	Filter	<none>
	Weight	<none>
	Split File	<none>
	N of Rows in Working Data File	19
Syntax		GRAPH /SCATTERPLOT(BIVAR)=Mean WITH Difference /MISSING=LISTWISE.
Resources	Processor Time	00:00:00.62
	Elapsed Time	00:00:01.00

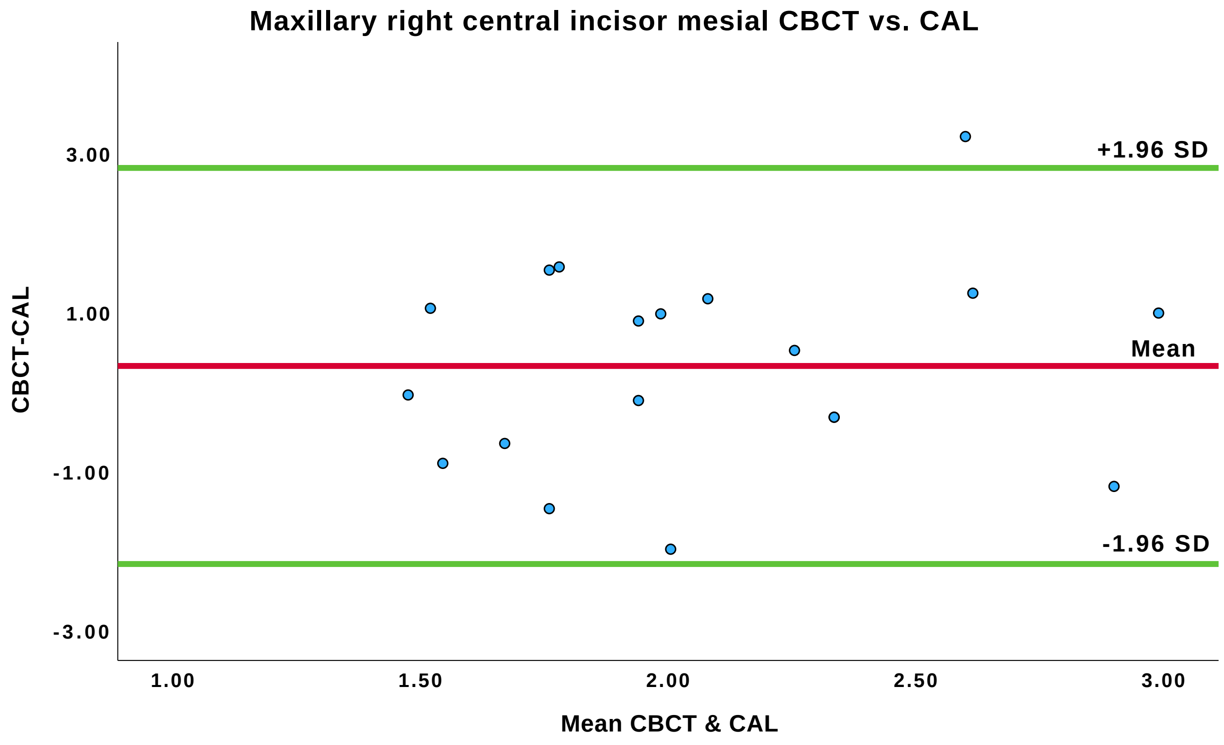

Supplement: S3 Appendix — (PDF) [file pone.0329604.s003.pdf]
